# Immune transcriptomes of highly exposed SARS-CoV-2 asymptomatic seropositive versus seronegative individuals from the Ischgl community

**DOI:** 10.1038/s41598-021-83110-6

**Published:** 2021-02-19

**Authors:** Hye Kyung Lee, Ludwig Knabl, Lisa Pipperger, Andre Volland, Priscilla A. Furth, Keunsoo Kang, Harold E. Smith, Ludwig Knabl, Romuald Bellmann, Christina Bernhard, Norbert Kaiser, Hannes Gänzer, Mathias Ströhle, Andreas Walser, Dorothee von Laer, Lothar Hennighausen

**Affiliations:** 1 Laboratory of Cell and Molecular Biology, National Institute of Diabetes, Digestive and Kidney Diseases, Bethesda, MD 20892 USA; 2 Institute of Hygiene and Medical Microbiology & Institute of Virology, Department of Hygiene, Microbiology and Public Health, Medical University of Innsbruck, 6020 Innsbruck, Austria; 3 Pathologie-Labor, 6020 Zams, Austria; 4 Departments of Oncology and Medicine, Lombardi Comprehensive Cancer Center, Georgetown University, Washington, District of Columbia USA; 5 Department of Microbiology, Dankook University, Cheonan, 31116 South Korea; 6 Krankenhaus St.Vinzenz Zams, 6511 Zams, Austria; 7 Clinical Pharmacokinetics Unit, Division of Intensive Care and Emergency Medicine, Department of Internal Medicine I, Medical University Innsbruck, 6020 Innsbruck, Austria; 8 Hospital Kufstein, 6330 Kufstein, Austria; 9 Bezirkskrankenhaus St. Johann in Tirol, 6380 St. Johann in Tirol, Austria; 10 Bezirkskrankenhaus Schwaz, 6130 Schwaz, Austria; 11 Intensive Care, Medical University of Innsbruck, 6020 Innsbruck, Austria; 12 Ordination (Private Practice), 6561 Ischgl, Austria

**Keywords:** Computational biology and bioinformatics, Immunology, Medical research

## Abstract

SARS-CoV-2 infection ranges from asymptomatic to severe with lingering symptomatology in some. This prompted investigation of whether or not asymptomatic disease results in measurable immune activation post-infection. Immune activation following asymptomatic SARS-CoV-2 infection was characterized through a comparative investigation of the immune cell transcriptomes from 43 asymptomatic seropositive and 52 highly exposed seronegative individuals from the same community 4–6 weeks following a superspreading event. Few of the 95 individuals had underlying health issues. One seropositive individual reported Cystic Fibrosis and one individual reported Incontinentia pigmenti. No evidence of immune activation was found in asymptomatic seropositive individuals with the exception of the Cystic Fibrosis patient. There were no statistically significant differences in immune transcriptomes between asymptomatic seropositive and highly exposed seronegative individuals. Four positive controls, mildly symptomatic seropositive individuals whose blood was examined 3 weeks following infection, showed immune activation. Negative controls were four seronegative individuals from neighboring communities without COVID-19. All individuals remained in their usual state of health through a five-month follow-up after sample collection. In summary, whole blood transcriptomes identified individual immune profiles within a community population and showed that asymptomatic infection within a super-spreading event was not associated with enduring immunological activation.

## Introduction

Coronavirus disease 2019 (COVID-19) is caused by severe acute respiratory syndrome coronavirus (SARS-CoV) 2. Clinical presentation ranges from asymptomatic to severe disease. In symptomatic patients, increased levels of pro-inflammatory cytokines and subsequently lymphopenia are reported coincident with disease^1,2^. Flow cytometry-based^3^ and single-cell transcriptome studies^4–7^ defined inflammatory milieus with an overt innate and adaptive immune activation and immune signatures of COVID-19 patients with different disease trajectories^7–10^.

On occasion COVID-19 patients can suffer from longer term sequelae. In one survey, thirty-five percent of patients with mild outpatient-treated disease were reported as having not returned to their usual state of health by 2–3 weeks following infection^11^. Prolonged myocardial inflammation^12^ and subacute thyroiditis^13^ post resolution of acute infection have been reported. Although it is increasingly being recognized that a large percentage of infected individuals are asymptomatic^14,15^, the response of their immune systems and prevalence of unrecognized ongoing inflammation to SARS-CoV-2 remains under investigation. This study was specifically designed to fill a gap in the understanding of immunological outcomes in asymptomatic individuals following a community super-spreading event. While other studies have focused on single cell RNA-seq (scRNA-seq) profiling small numbers of individuals with COVID-19 disease^16^, the use of RNA-seq from peripheral blood mononuclear cells (PBMCs) permits the analysis of larger cohorts at a great depth. Whole blood transcriptomes for immune characterization have been used previously in studies of symptomatic tuberculosis and COVID-19 patients^17,18^. Moreover, with the pandemic extending itself into populations with underlying genetic disease, there is also an urgent need to understand their immune response and immunity to SARS-CoV-2 infections. Here, our community-based study included one asymptomatic seropositive patient with cystic fibrosis (CF) and one with Nuclear factor-kappa B Essential Modulator (NEMO) deficiency.

Here, using blood transcriptomes from the Ischgl community that had experienced a super spreading event, we provide evidence that asymptomatic infection can resolve without evidence of immunological activation 4–6 weeks following infection. Our data contributes to ongoing large-scale efforts by many labs to understand the spectrum of immune response elicited by SARS-CoV-2 infection. Limitations of the study include a population largely with few underlying health issues and a modest number of samples, albeit relatively evenly matched between seropositive and seronegative individuals, and reflecting over 5% of the total Ischgl community.

## Results

In an attempt to define the immune response in asymptomatic SARS-CoV-2 seropositive and highly exposed seronegative individuals within an isolated community population, we conducted a study on residents from the ski resort of Ischgl that experienced a superspreading event in early March of 2020. This explosive local outbreak led to the spread of the virus throughout Austria and to many other European countries and worldwide^19^. Ischgl and the Paznaun valley were quarantined on March 13, 2020 and remained under lockdown for 6 weeks. An epidemiologic study targeting 79% (n = 1473) of the population of Ischgl (n = 1867)^20^ was conducted between April 21 and 27 using the SARS-CoV-2 RT-PCR kit, antibody tests and WHO guidelines and revealed a seroprevalence of approximately 42% with approximately 17% of these being asymptomatic.

**Figure 1:**
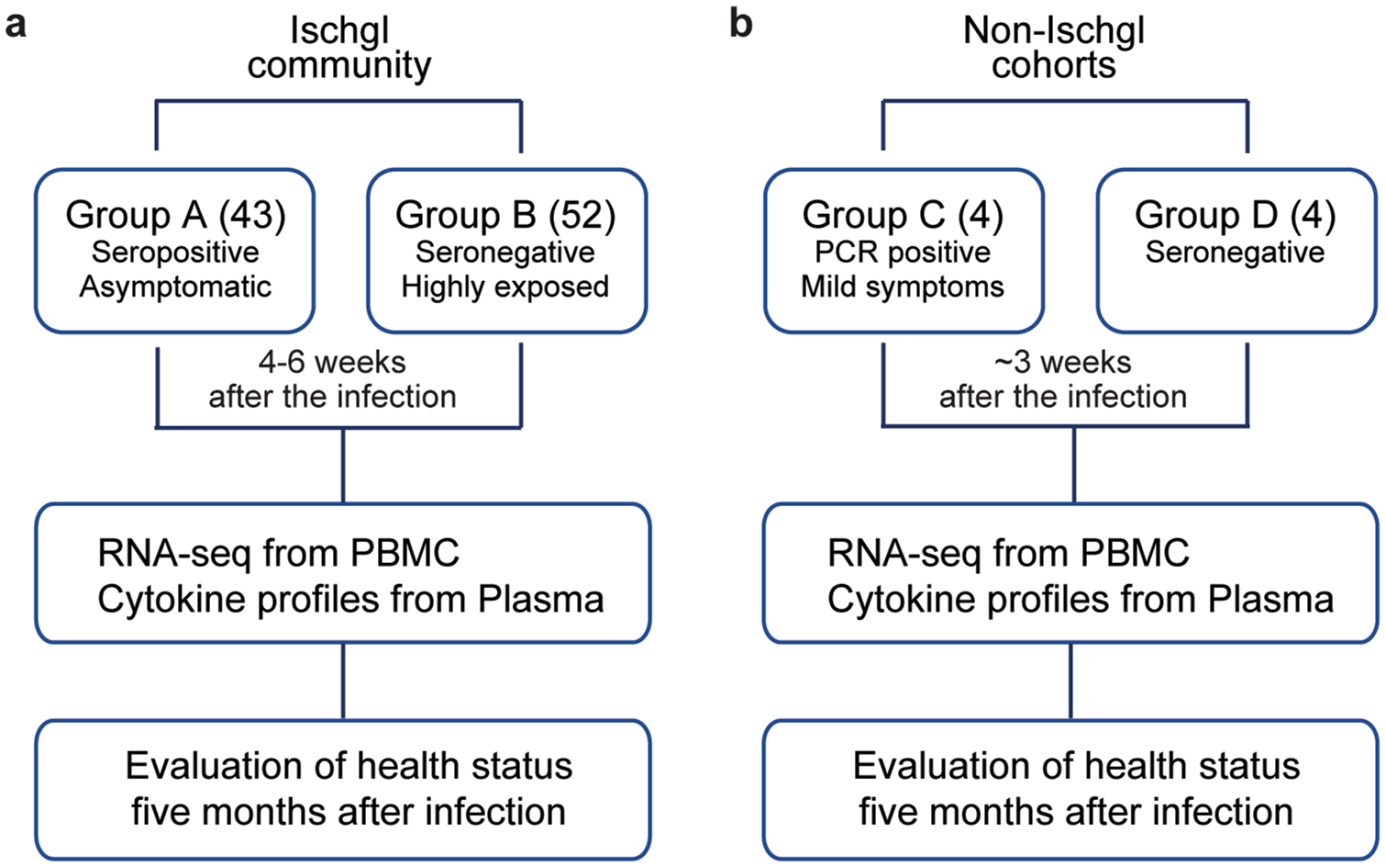
Study design. **a** In the Ischgl community, 43 seropositive asymptomatic and 52 highly exposed seronegative individuals underwent phlebotomy and health evaluation between April 21 and 27, 2020 with a follow up health evaluation mid-August, 2020. The SARS-CoV-2 superspreading event occurred in the community between the end of February and March 13 of 2020 when the town was quarantined. **b** The non-Ischgl cohorts were recruited from other parts of Tyrol. Four SARS-CoV-2 PCR positive patients exhibiting mild symptoms, who underwent phlebotomy and health evaluation approximately 3 weeks after being confirmed as PCR positive for SARS-CoV-2 and four seronegative individuals from a neighboring community without COVID-19. Figures were generated using Adobe Illustrator 2020 for Mac OS X.

We systematically investigated the host immune response of asymptomatic seropositive patients, asymptomatic seronegative patients, positive and negative controls (Supplementary Table 1) through whole blood transcriptomes by RNA-sequencing (RNA-seq). All participants were interviewed about their contact to COVID-19 positive individuals, their clinical symptoms and positive SARS-CoV-2 tests in the past. Furthermore, all participants were screened for anti-SARS-CoV-2 antibodies by using three different assays, a neutralizing antibody assay, an anti-SARS-CoV-2-S1-protein assay and an anti-SARS-CoV-2-N-protein IgG assay. For analysis, samples were divided into four groups: A. Seropositive, asymptomatic defined as samples from antibody-positive participants without history of COVID-19 symptoms (n = 43); B. Seronegative, highly exposed defined as samples from seronegative participants, some of which lived in the same household with COVID-19 positive individuals, without any infection control measures (n = 52); C. Positive controls defined as samples from seropositive, mildly symptomatic individuals taken 3 weeks after infection (n = 4); and D. Negative controls defined as samples from seronegative individuals without any contact to SARS-CoV-2 positive individuals (n = 4) (Fig. 1).

**Figure 2:**
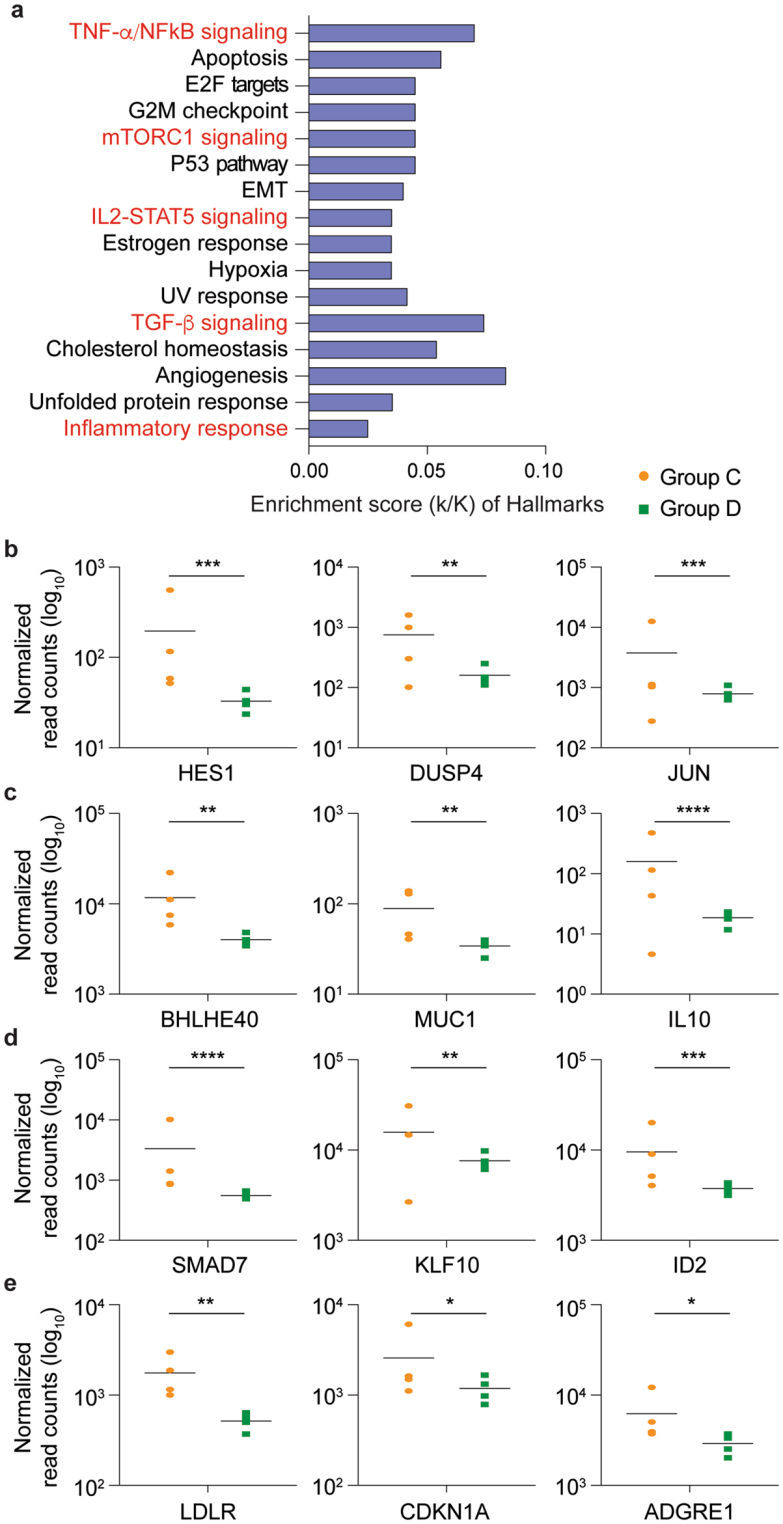
SARS-CoV-2 infected patients with mild symptoms demonstrated significant elevations in immune response genes 3 weeks following PCR confirmation of SARS-CoV-2. **a** Genes expressed at significantly higher levels in the four infected patients were significantly enriched in 16 Hallmark Gene Sets (FDR q value < 0.006). Five are involved in immune regulation: TNF-a NFκB, mTORC1, IL2-STAT5, TGFβ, inflammatory response (labeled in red). The other 11 gene sets are not directly linked to immune response. **b–e** Comparison of relative normalized gene expression levels of three representative genes from each of the four immune regulation-related Hallmark Gene Sets between infected (orange dots, Group D, n = 4) and non-infected (green dots, Group E, n = 4) individuals. Mean indicated. *Padj < 0.04, **Padj < 0.001, ***Padj < 0.0001, ****Padj < 0.00001 (DESeq2). Figures were generated using GraphPad PRISM version 8.2 and Adobe Illustrator 2020 for Mac OS X.

Positive controls (Group C) and negative controls (Group D) were used to validate that an unbiased RNA-seq analysis performed on RNA extracted from peripheral blood mononuclear cells (PBMCs) could be used to identify gene expression changes in patients following SARS-CoV-2 infection (Figs. 1b and 2, and Supplementary Table 2). Among the 175 genes whose expression was significantly elevated in PBMCs from seropositive symptomatic patients, genes were significantly enriched in 16 Hallmark gene sets, five of which have defined roles in immune regulation: TNF-α/NFκB, mTORC1 signaling, IL2-STAT5 signaling, TGFβ signaling and inflammatory response. This demonstrated that the RNA-seq approach utilizing buffy-coat-isolated PBMCs identified statistically significant differences in inflammatory gene expression between infected and non-infected individuals. Notably, we observed significantly elevated *IL10* expression in the seropositive, symptomatic patients, a cytokine whose elevated expression has been associated with COVID-19 disease severity^21^.

**Table 1:** Demographic and clinical characteristics of asymptomatic SARS-CoV-2 seropositive and highly exposed seronegative individuals test (Chi-Square 7.66, p-value 0.36).

Group	Group A				Group B			
	Seropositive, asymptomatic				Seronegative, highly exposed			
Total number	43				52			
Age	1–20	21–40	41–63	> 64	1–20	21–40	41–63	> 64
Number	8	16	17	2	17	11	19	5
**Sex**								
Male	5	11	11	2	10	4	8	4
Female	3	5	6	0	7	7	11	1
Risk factors associated with severe COVID19 disease*								
**Diabetes**								
Male								1
Female							1	
Hypertension			5	1			1	2
Serious heart conditions			1					
Cystic fibrosis		1						
Chronic kidney disease		1						
Liver disease		1						
**Cerebrovascular disease (affects blood vessels and blood supply to the brain)**								
Immunocompromised state		2					1	
Cancer	1	1						
**Obesity (BMI > 30)**								
Male	2	1	1				2	
Female			1				1	
Patients reporting new symptoms post exposure (fatigue, lung or cardiac SOB)	None	None	None	None	None	None	None	None

**Figure 3:**
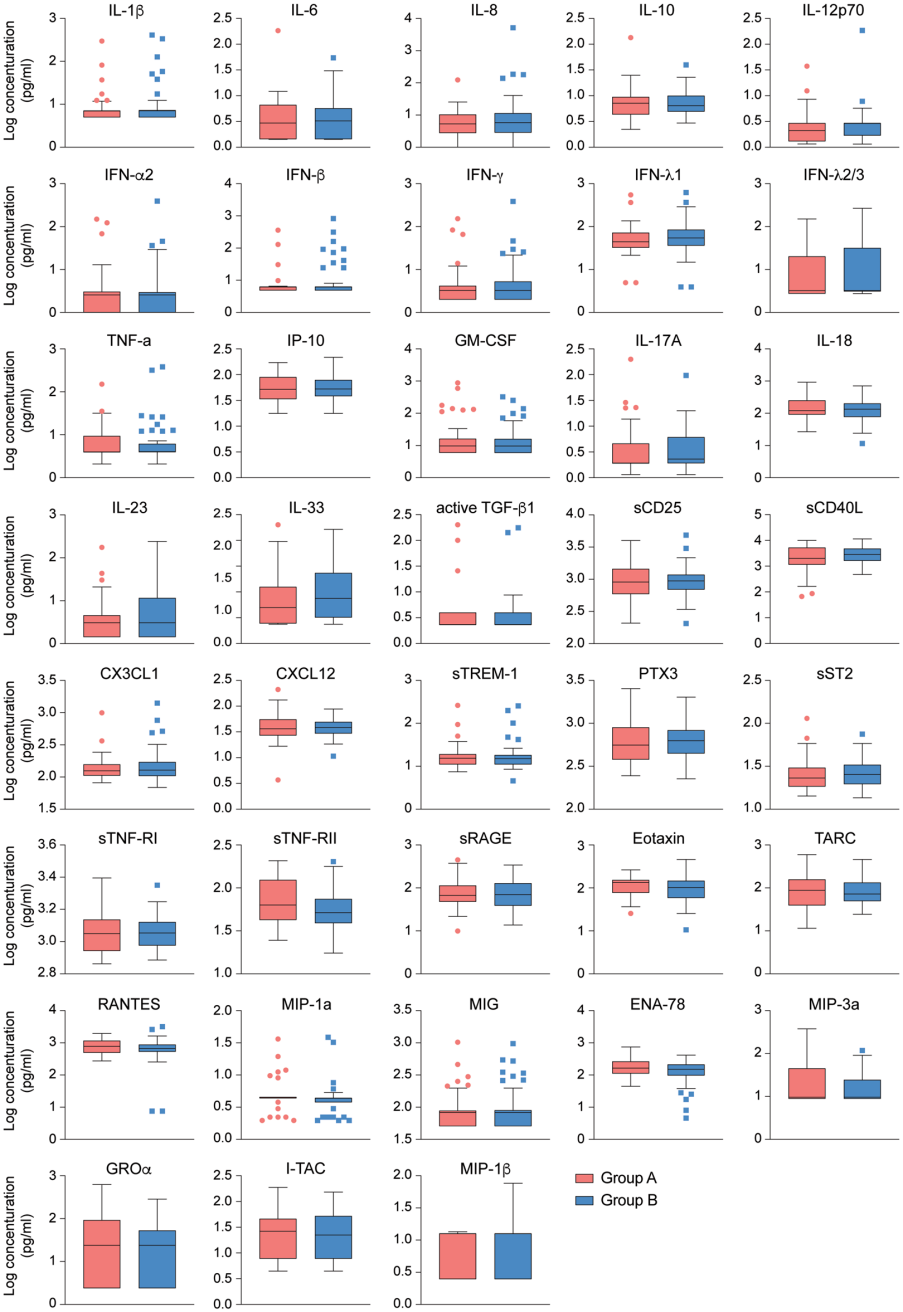
Comparison of cytokine and chemokine levels between asymptomatic seropositive and highly exposed seronegative cohorts. No statistically significant differences in relative steady state protein levels of 28 cytokines and 10 chemokines between asymptomatic seropositive (red, Group A, n = 41) and highly exposed seronegative (blue, Group B, n = 52) were found. Relative protein levels measured using LEGENDplex assays. Boxplots show median (middle bar), interquartile range (IQR) (box), 1.5 X IQR (whiskers). Figures were generated using GraphPad PRISM version 8.2 and Adobe Illustrator 2020 for Mac OS X.

Next, unbiased RNA-seq analyses were performed on RNA extracted from PBMCs from the 43 seropositive asymptomatic individuals (Group A) and 52 highly exposed seronegative individuals (Group B) (Fig. 1a, Supplementary Table 1). The gender and age distribution between the two groups was similar (Table 1. Chi-Square p > 0.05). Six households had both seropositive asymptomatic and highly exposed seronegative members (Supplementary Table 1). Two asymptomatic seropositive individuals had conditions that theoretically could impact immune activation and/or response to COVID-19^22^, one individual with Cystic Fibrosis *(CFTR* G551D mutation) and one individual with Nuclear factor-kappa B Essential Modulator (NEMO) deficiency (Incontinentia pigmenti, *IKBKG* exon4_10del mutation) (Table 1). Seropositive asymptomatic individuals were asymptomatic throughout the time of the super-spreading event, through the time of blood collection (4–6 weeks after the super-spreading event), and remained so 5 months following the super-infection, confirmed with individual phone calls to study participants. Very few statistically significant changes in gene expression were found between asymptomatic, seropositive individuals and highly exposed, seronegative individuals (11 induced, 7 downregulated) (Supplementary Table 3). Plasma cytokine profiling similarly revealed no significant differences (Fig. 3 and Supplementary Table 4).

**Figure 4:**
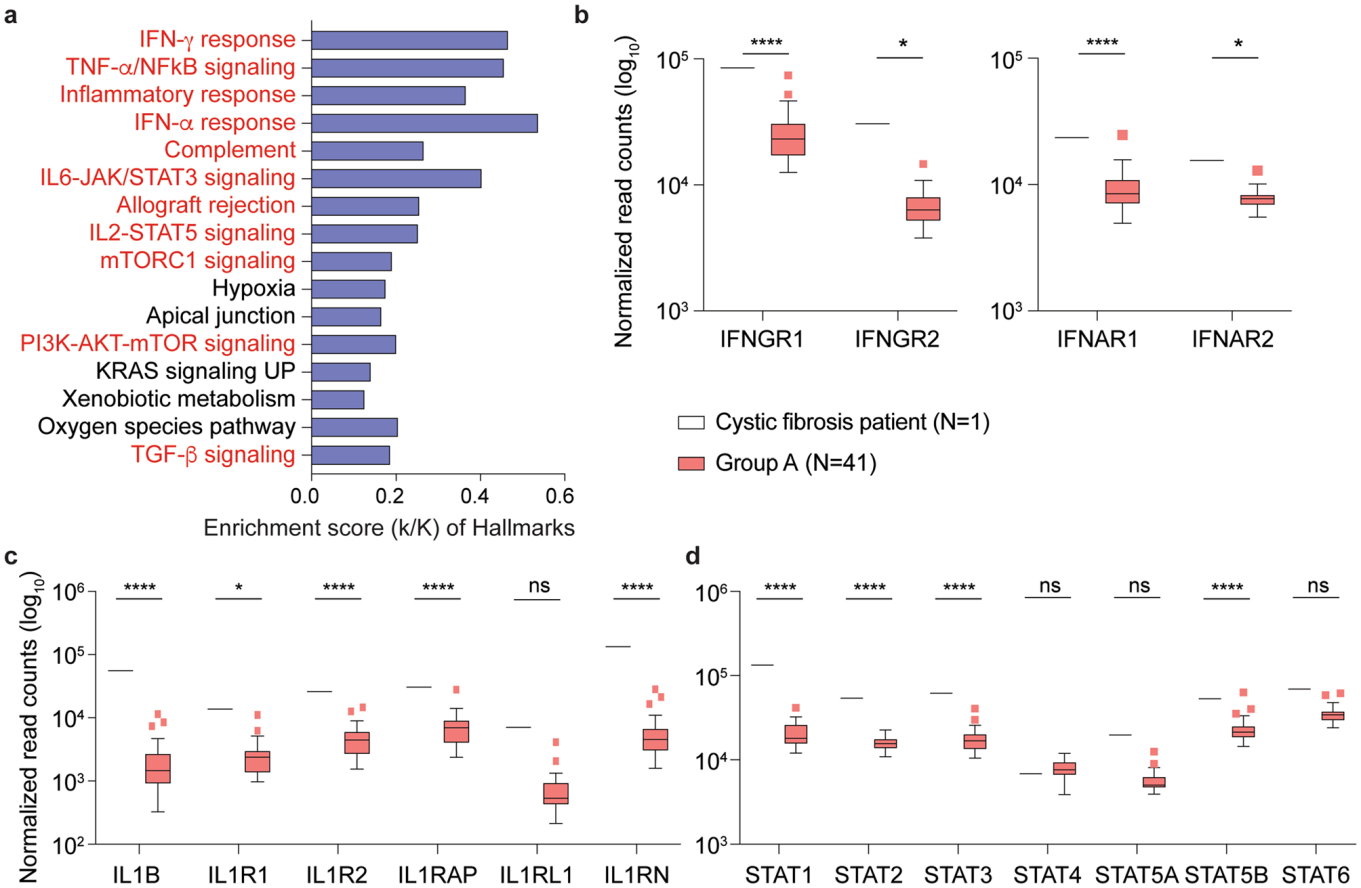
Comparison of gene expression levels between the Cystic Fibrosis patient and the remainder of the asymptomatic seropositive cohort. **a** Genes expressed at significantly higher levels in the Cystic Fibrosis patient were significantly enriched in Hallmark Gene Sets (FDR q value < 0.005). Out of the top 16, eleven are involved in immune regulation: IFNγ, TNFα via NFκB, inflammatory response, IFNα, complement, IL6-JAK/STAT3, allograft rejection, IL2-STAT5, mTORC1, PI3-AKT-mTOR, TGFβ (labeled in red). The other five gene sets are not directly linked to immune response (labeled in black). **b–d** Comparison of relative normalized gene expression levels from IFNγ (IFNGR1, IFNGR2), IFNα (IFNAR1, IFNAR2) and TNFα inflammatory signal (IL1B, ILR1, ILR2, IL1RAP, IL1RL1, ILRN) response and STAT family genes (STAT1, STAT2, STAT3, STAT4, STAT5A, STAT5B and STAT6). Boxplots show median (middle bar), interquartile range (IQR) (box), 1.5 X IQR (whiskers). *P < 0.05, ****P < 0.00001, ns, not significant. Figures were generated using GraphPad PRISM version 8.2 and Adobe Illustrator 2020 for Mac OS X.

Neither of the two individuals with genetic disorders, one female with cystic fibrosis (CF) and one female with *Incontinentia pigmenti* (NEMO), both part of the asymptomatic seropositive group, were on ongoing medical therapy during the time of the study. The CF patient demonstrated significant differences in expression for approximately 4670 genes (Supplementary Table 5). Overall, expression levels of 3020 genes were significantly higher and expression levels of 1648 genes were significantly lower. Genes were enriched in 32 Hallmark gene sets, 11 of which have defined roles in immune regulation (Fig. 4a). Expression of key immune signature genes, including interferon response genes, IL1B, IL17A and their respective receptors, and JAK-STAT pathway genes, were significantly induced (Fig. 4b–d). In contrast, the NEMO patient showed no significant immune transcriptome differences as compared to other asymptomatic, seropositive individuals (Supplementary Table 6).

## Discussion

The global spread of SARS-CoV-2 calls for a thorough molecular understanding of the pathophysiology of the disease. Dysregulation of immune responses have been implicated in large spectrum of disease manifestation and our whole blood transcriptomic analysis provides evidence that asymptomatic infection can resolve without evidence of prolonged immunological activation. Furthermore, our study demonstrates that whole blood transcriptome experiments are a valuable tool to monitor immune profiles in community-based cohorts. In contrast to the increasingly popular single cell RNA-seq approaches^16,23^, whole blood transcriptomes can be easily conducted in large clinical cohort studies, providing in depth molecular information for significant proportions of the population.

The ability to assess the immune response of both symptomatic and asymptomatic SARS-CoV-2 infected individuals provides critical information on dysregulated immune-response signatures that might foretell disease trajectories. While longitudinal studies on hospitalized patients demonstrate elevated levels of pro-inflammatory cytokines and signatures associated with ongoing inflammation^3^, there is a parallel need to pinpoint the immune response of infected, yet asymptomatic individuals. With the exception of a CF patient, we did not detect an aberrant immune transcriptome in 43 asymptomatic seropositive individuals that were infected during a super spreading event as compared to 52 highly exposed seronegative individuals from the same community, with some seropositive and seronegative individuals coming from the same household. These results demonstrate that development of an antibody response to COVID-19 following viral exposure and seroconversion in asymptomatic cases is not necessarily associated with sustained alterations in the immune system transcriptome.

A previous study of 37 COVID-19 test-positive asymptomatic individuals in the Wanzhou District^24^ reported similar levels of virus-specific IgG but reduced neutralizing antibody compared to symptomatic patients coupled with lower levels of inflammatory cytokines in the asymptomatic individuals. In contrast, in our much larger seroprevalence study for Ischgl population (n = 1867)^20^, the amounts of anti-SARS-CoV-2 and neutralizing antibody were measured in individuals serum using ELISA. Here we found that virus-specific IgG levels coincided with the values of neutralizing antibody in the 42.2% of the individuals that were asymptomatic and seropositive.

While other studies have focused on single cell RNA-seq (scRNA-seq) profiling of small numbers of individuals with COVID-19 disease^4–7,9,10^, the use of RNA-seq from mononuclear cells permitted us to analyze larger cohorts at a great depth. This approach was validated through the detection of inflammatory immune signatures in COVID-19 patients exhibiting mild symptoms and one asymptomatic seropositive CF patient.

Patients with CF manifest cytokine dysfunction and hyperinflammation that overlaps with the pathophysiology of COVID-19^25^. While there are limited data on the immune response of CF patients to COVID-19 infection, preliminary information suggests that the course of disease may be milder than expected^26,27^. The immune transcriptome of the asymptomatic seropositive CF patient provided evidence of highly activated cytokine signaling pathways. Expression of key components of interferon, interleukin and JAK-STAT pathways is highly elevated. Many of these genes, such as IL1B and its receptors are also highly activated in COVID-19 patients^9^. In contrast, the immune transcriptome of a SRAS-CoV-2 infected asymptomatic patient with a NEMO deficiency syndrome, a rare primary immunodeficiency, was indistinguishable from asymptomatic seropositive controls. It remains to be understood how elevated cytokine signaling, documented here in an asymptomatic CF patient, contributes to disease progression in non-CF patients and why CF patients, with chronic high expression, do not invariably experience disease progression. A critical question for development of targeted therapies is to discern between direct pathogenic immune determinants of severe disease and correlates of inflammation. As the sample size of seropositive patients with genetic disease is too small, to draw definitive conclusions about the aberrant immune profile obtained for the CF patient, a follow up study with more patients would be required.

Limitations of our study include the observation that there were few underlying health issues in this rural alpine population living at an altitude of 1400 m above sea level. For example, obesity, which is associated with an inflammatory state and is recognized as risk factor for severe COVID-19 disease^28,29^, was less than 10% in our study population, differing greatly from higher prevalence rate in other infected populations. Another limitation is that we had only single examples of known individuals with genetic mutations associated with disease (one CF patient and one NEMO patient). Finally, available funding limited RNA-seq transcriptome profiling to 100 individual samples.

## Methods

### Study population, study design and recruitment

Recruitment and blood sample collection took place between April 21 and 27, 2020. This study was approved by the Institutional Review Board (IRB) of the Office of Research Oversight/Regulatory Affairs, Medical University of Innsbruck, Austria (EC numbers: 1100/2020 and 1111/2020). A waiver of informed consent was obtained from the Institutional Review Board (IRB) of the Office of Research Oversight/Regulatory Affairs, Medical University of Innsbruck. This study was determined to impose minimal risk on participants. All methods were carried out in accordance with relevant guidelines and regulations. All research has been have been performed in accordance with the Declaration of Helsinki (https://www.wma.net/policies-post/wma-declaration-of-helsinki-ethical-principles-for-medical-research-involving-human-subjects/).

This cross-sectional epidemiological survey targeted all residents of Ischgl/Tyrol irrespective of age and gender. We followed the ‘Sex and Gender Equity in Research – SAGER – guidelines’ and included sex and gender considerations where relevant.

At the time of investigation, Ischgl had a population size of 1867 individuals, 1617 with their main residence in Ischgl and 250 seasonal immigrant workers, living in 582 different households. COVID-19 diagnosis was done using the RealStar SARS-CoV-2 RT-PCR kit 1.0 (Altona Diagnostics GmbH, Hamburg, Germany), the Anti-SARS-CoV-2 ELISA (IgA) and the Anti-SARS-CoV-2 ELISA (IgG) (Euroimmun, Lübeck, Germany) and the SARS-CoV-2 immunoassay (Abbott, IL, USA). Each group was categorized by assay results interpreted by the WHO guidelines.

Due to strict lockdown-restrictions at the time of sampling, the time of infection of seropositive asymptomatic individuals can be assumed to be within the same timeframe as other community members, 4–6 weeks prior to sample collection. Asymptomatic seropositive individuals were all from households with identified SARS-CoV-2 infected individuals as well as six of the 52 seronegative individuals. The COVID-19 diagnosis of household members was confirmed either by PCR testing or by clinical diagnosis plus evaluation of antibodies against SARS-CoV-2.

### Ethical approval

This study was approved by the Institutional Review Board (IRB) of the Office of Research Oversight / Regulatory Affairs, Medical University of Innsbruck, Austria (EC numbers: 1100/2020 and 1111/2020).

### Quantification of immunoproteins

Samples from all groups were measured for the presence and quantity of several human inflammatory cytokines/chemokines, key targets, which are involved in inflammation and immune response and human proteins that are involved in response to viral infections. For this purpose, participants’ plasma was examined by using four different pre-defined LEGENDplex assays (BioLegend, CA), while the human inflammation panel 1 was used to quantify 13 human inflammatory cytokines including IL-17A, IL-18, IL-23 and IL-33, the pre-defined human inflammation panel 2 quantified TGF-β1, sTREM1, PTX-3, sCD40L, sCD25, CXCL12, sST2, sTNF-RI, sTNF-RII, sRAGE and CX3CL1. The human anti-virus response panel measured quantities of type 1 interferons (INF-α2, IFN-β), type 2 interferons (IFN-γ), and type 3 interferons (IFN-λ1, IFN-λ2/3) as well as the interleukins IL-1β, IL-6, IL-8, IL-10, IL-12 and TNF-α, IP-10 and GM-CSF. Additionally, chemokines were analyzed using the human proinflammatory chemokine panel. The LEGENDplex assays were used according to the manufacturer’s instructions. In short, immunoprotein capturing by antibody-bearing beads was done by using V-bottom plates. Bead-bound proteins were measured with a FACS CantoII cytometer (Becton Dickinson, NJ), whereby fluorescent signal intensity was proportional to the amount of bead-bound proteins. The concentrations of the immunoproteins were calculated with the LEGENDplex data analysis software.

### Extraction of the buffy coat and purification of RNA

Extraction of the buffy coat and subsequent RNA purification will be performed as described^10^. In short, the drawn blood is centrifuged at 1600*g* for 10 min at 4 °C. After vacuming off the plasma layer, the buffy coat layer is carefully collected. The obtained buffy coat is mixed with 1 mL RBC lysis buffer and incubated for 10 min at room temperature. After a centrifugation step, supernatants were discarded, and the pellets mixed with 1 mL RBC lysis buffer. The pellet is washed with PBS buffer and then mixed with 1 mL TRIzol reagent. For extraction of the RNA the TRIzol reagent single-step method will be used^11^. After addition of 2 M sodium acetate (pH 4), the tube is mixed thoroughly by invertion before adding chloroform/isoamyl alcohol (49:1) and another mixing step. The sample gets incubated on ice for 15 min and subsequently centrifuged for 20 min at 10,000*g* and 4 °C. The obtained aqueous phase is transferred to a new Eppendorf tube, 1 mL isopropanol is then added, which is followed by an incubation at − 20 °C for 1 h to precipitate RNA. The final RNA precipitate is won by centrifugation at 10,000*g* at 4 °C and discarding of the supernatant. Pellets are stored at − 80 °C until dispatch.

### mRNA sequencing (mRNA-seq) and data analysis

Nanophotometer (Implen) was used to analyze each sample for concentration and the RNA quality was assessed by an Agilent Bioanalyzer 2100 (Agilent Technologies, CA). The Poly-A containing mRNA is purified by poly-T oligo hybridization from 1 μg of total RNAs and cDNA was synthesized using SuperScript III (Invitrogen, MA). Libraries for sequencing were prepared according to the manufacturer’s instructions with TruSeq Stranded mRNA Library Prep Kit (Illumina, CA, RS-20020595) and paired-end sequencing was done with a NovaSeq 6000 instrument (Illumina).

The raw data were subjected to QC analyses using the FastQC tool (version 0.11.9) (https://www.bioinformatics.babraham.ac.uk/projects/fastqc/). mRNA-seq read quality control was done using Trimmomatic^30^ (version 0.36) and STAR RNA-seq^31^ (version STAR 2.5.4a) using 150 bp paired-end mode was used to align the reads (hg19). HTSeq^32^ (version 0.9.1) was to retrieve the raw counts and subsequently, R (https://www.R-project.org/), Bioconductor^33^ and DESeq2^34^ were used. Additionally, the RUVSeq^35^ package was applied to remove confounding factors. The data were pre-filtered keeping only those genes, which have at least ten reads in total. The visualization was done using dplyr (https://CRAN.R-project.org/package=dplyr) and ggplot2^36^. Genes were categorized as significantly differentially expressed with an adjusted p value (pAdj) below 0.05 and a fold change > 2 for up-regulated genes and a fold change of < − 2 for down-regulated ones. The genes were cutoff in the standard, less than 5 value and less than 1 log_2_ fold change and then conducted gene enrichment analysis (https://www.gsea-msigdb.org/gsea/msigdb).

### Statistical analysis

Due to the non-normality of cytokines as examined a normal distribution using the Kolmogorov–Smirnov test, the cytokine values obtained were log-transformed. Differences of log-transformed cytokines between two groups were examined using the Wilcoxon rank sum test corrected for multiple testing by the Benjamini-Krieger-Yekutieli method. P value was summarized in Supplementary Table 4. All data is presented as boxplots show median (middle bar), interquartile range (IQR) (box), 1.5 X IQR (Tukey whiskers) using GraphPad Prism 8 software (version 8.2.0). For comparison of RNA expression levels between CF patient and asymptomatic seropositive cohort, a two-way ANOVA followed by Tukey’s multiple comparisons test was used (GraphPad PRISM version 8.2 for Mac OS X, GraphPad Software). *P* < 0.05 was considered statistically significant.

## Supplementary Information

Supplementary Legends.

Supplementary Table 1.

Supplementary Table 2.

Supplementary Table 3.

Supplementary Table 4.

Supplementary Table 5.

Supplementary Table 6.

## Data Availability

The RNA-seq data of patients were uploaded to GSE162562. We confirm that all methods were carried out in accordance with relevant guidelines and regulations. Figures were generated using Adobe Illustrator 2020 for Mac OS X.
