# Supplementary material for: Immune transcriptomes of highly exposed SARS-CoV-2 asymptomatic seropositive versus seronegative individuals from the Ischgl community
Source: Sci Rep. 2021 Feb 19;11:4243. doi: 10.1038/s41598-021-83110-6 (PMC7895922; doi:10.1038/s41598-021-83110-6)
Supplement: Supplementary file 1 — Supplementary Legends. [file 41598_2021_83110_MOESM1_ESM.pdf]

Immune transcriptomes of highly exposed SARS-CoV-2 asymptomatic seropositive versus seronegative individuals from the Ischgl community

Hye Kyung Lee[1,*], Ludwig Knabl[2,*,†], Lisa Pipperger[2], Andre Volland[2], Priscilla A. Furth[3], Keunsoo Kang[4], Harold E. Smith[1], Ludwig Knabl Sr.[5], Romuald Bellmann[6], Christina Bernhard[7], Norbert Kaiser[8], Hannes Gänzer[9], Mathias Ströhle[10], Andreas Walser[11], Dorothee von Laer[2] and Lothar Hennighausen[1,†]

[1]National Institute of Diabetes, Digestive and Kidney Diseases, Bethesda, MD 20892, USA; [2]Institute of Virology, Department of Hygiene, Medical Microbiology and Public Health, Medical University of Innsbruck, Austria; [3]Departments of Oncology & Medicine, Georgetown University, Washington, DC, USA; [4]Dankook University, Cheonan, South Korea; [5]Krankenhaus St.Vinzenz Zams, Austria; [6]Medical University Innsbruck, Innsbruck, Austria; [7]Hospital Kufstein, Austria; [8]Bezirkskrankenhaus St. Johann in Tirol, Austria; [9]Bezirkskrankenhaus Schwaz, Austria; [10]Intensive Care, Medical University of Innsbruck, Austria; [11]Ordination (Private Practice), Ischgl, Austria.

[*] Equal contribution
[†] Correspondence to: L.K. (Ludwig.knabl@i-med.ac.at) and L.H. (lotharh@niddk.nih.gov)

Supplementary Tables

Supplementary Table 1. Grouping and household of tested patients.

Supplementary Table 2. A list of genes and GSEA analysis for mild symptomatic and seronegative patients of non-Ischgl residents (Group D and E).

Supplementary Table 3. Gene list and GSEA analysis for asymptomatic and seronegative patients (Group A and B).

Supplementary Table 4. Cytokine profiles of all individuals.

Supplementary Table 5. Gene expression profiles and GSEA analysis from the cystic fibrosis patient and asymptomatic patients (Group A).

Supplementary Table 6. Gene expression profiles and GSEA analysis from the NEMO deficient patient and asymptomatic patients (Group A).
